# Sex Biased Gene Expression Profiling of Human Brains at Major Developmental Stages

**DOI:** 10.1038/srep21181

**Published:** 2016-02-16

**Authors:** Lei Shi, Zhe Zhang, Bing Su

**Affiliations:** 1 Faculty of Life Science and Technology, Kunming University of Science and Technology, Kunming 650500, China; 2 State Key Laboratory of Genetic Resources and Evolution, Kunming Institute of Zoology, Chinese Academy of Sciences, Kunming 650223, China; 3 Yunnan Key Laboratory of Primate Biomedical Research, Kunming 650000, China; 4 The Molecular & Behavioral Neuroscience Institute (MBNI), University of Michigan, 109 Zina Pitcher Place, Ann Arbor, MI 48109-2200, USA

## Abstract

There are many differences in brain structure and function between males and females. However, how these differences were manifested during development and maintained through adulthood are still unclear. Here we present a time series analyses of genome-wide transcription profiles of the human brain, and we identified genes showing sex biased expression at major developmental stages (prenatal time, early childhood, puberty time and adulthood). We observed a great number of genes (>2,000 genes) showing between-sex expression divergence at all developmental stages with the greatest number (4,164 genes) at puberty time. However, there are little overlap of sex-biased genes among the major developmental stages, an indication of dynamic expression regulation of the sex-biased genes in the brain during development. Notably, the male biased genes are highly enriched for genes involved in neurological and psychiatric disorders like schizophrenia, bipolar disorder, Alzheimer’s disease and autism, while no such pattern was seen for the female-biased genes, suggesting that the differences in brain disorder susceptibility between males and females are likely rooted from the sex-biased gene expression regulation during brain development. Collectively, these analyses reveal an important role of sex biased genes in brain development and neurodevelopmental disorders.

Sexual dimorphism is a common phenomenon in humans and many non-human primate species, such as chimpanzee and rhesus macaque. It is exhibited in a variety of physical characteristics, such as body size, hair color and skeletal structure, as well as structure and function of central nervous system (CNS)^1^. For example, structural connectome analysis of the human brain showed that male brains are structured to facilitate connectivity between perception and coordinated actions, whereas female brains are designed to facilitate communication between analytical and intuitive processing modes^2^,^3^,^4^. In view of disease, many CNS disorders show sex differences in their incidence and/or nature, including but not limited to Alzheimer’s disease (AD), schizophrenia, autism, addiction and attention deficit disorder^4^. AD represents a striking example of between-sex difference. Up to 90% of older men show AD-related neurofibrillary pathology, whereas it is found in only 8–10% of age-matched women^4^. Therefore, understanding the molecular basis of the known sex differences of the brain is of obvious importance to understanding human cognition and human mental disorders.

Evolutionary analysis of the sex biased genes showed that these genes, especially the male biased genes tend to evolve rapidly in protein sequences^5^. For example, in fruit fly, genes with male biased expression are more functionally divergent between species than those with female biased expression^6^. This pattern is also exhibited in nematodes^7^, as well as in mammals (mouse and rat) during spermatogenesis^8^. Comparison of human and chimpanzee genes especially those genes expressed in testis showed that the male biased genes showed rapid evolution among multiple primate lineages^9^. Analysis of primate occipital cortex gene expression also showed that the coding regions of the female biased genes are more evolutionarily constrained compared to those of the male biased genes^10^. Recent studies found that evolution of sex biased genes are developmental stage dependent with sex specific selection pressures, revealing previously unknown complexity^11^,^12^.

Recently, global transcription profiling has revealed large proportions of the genome with markedly different expression levels in males and females^13^,^14^,^15^. However, these studies focused almost exclusively on the adult human brains with only a few studies on prenatal stages^16^, and the ontogenetic complexity of sexual dimorphism and the sex-biased genes remain largely unexplored. In addition, all previous studies were based on microarray data with limited genome coverage. Fortunately, due to the next generation sequencing technology, we now can explore human ontogenetic complexity of sexual dimorphism and sex biased genes by analyzing a large amount of high quality RNA-seq data with deep genome coverage.

In this study, we compared the brain transcriptomes of males and females at four major developmental stages, *i.e.* prenatal period, early childhood, puberty time and adulthood. Totally, 14–15 distinct brain regions were analyzed. We conducted functional annotation of the identified sex biased genes at different developmental stages. Our results indicated that sex biased gene expression through human ontogeny is more complex than previously revealed.

## Results

### Identification of spatiotemporal sex biased genes in the human brain

In order to understand the expression changes of the brain sex biased genes in a spatiotemporal manner, we divided the brain development into four major stages, including prenatal time, early childhood, puberty time and adulthood (Table 1). The transcriptome data of more than 14 brain regions were analyzed (Supplementary Table 1–8).

**Figure 1 f1:**
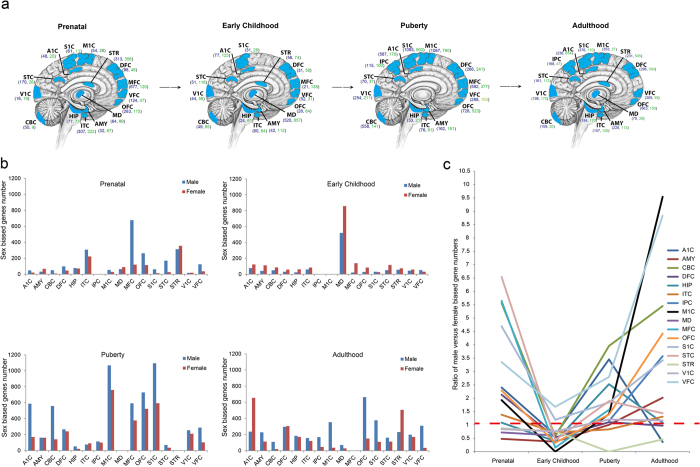
Identification of spatiotemporal sex biased genes in the human brain^44^. (**a**) Number of sex biased genes (p < 0.05, negative binomial test) within each brain region during human brain development. Dark blue numbers indicate male biased genes; Green numbers indicate female biased genes; (**b**) Histogram plot of number of sex biased genes of each brain region during human brain development; (**c**) Line graph of ratio of male sex biased genes vs. female biased genes. The studied brain regions include A1C (primary auditory cortex), S1C (primary somatosensory cortex), M1C (primary motor cortex), STR (striatum), DFC (dorsolateral prefrontal cortex), MFC (medial prefrontal cortex), VFC (ventrolateral prefrontal cortex), OFC (orbital frontal cortex), MD (mediodorsal nucleus of thalamus), AMY (amygdaloid complex), ITC (inferolateral temporal cortex), HIP (hippocampus), CBC (cerebellar cortex), V1C (primary visual cortex), STC (posterior(caudal) superior temporal cortex) and IPC (posterior inferior parietal cortex).

Genes with sex biased expression (*p* < 0.05, negative binomial test) were investigated in each brain region at different developmental stages and the results are summarized in Fig. 1. We found that in prenatal time, most of the brain regions have more male biased genes compared with the female-biased genes except for four brain regions (AMY, MD, STR and V1C) (Fig. 1a[Fig f1]b), suggesting that the brain transcriptome bias between males and females starts at prenatal stage and is mainly driven by the male-biased genes^17^. In contrast, during childhood, the pattern changes with more female-biased genes in most of the brain regions except for S1C and VFC. At puberty time, the male-biased genes become dominant again for most of the brain regions. In contrast, no general trend is observed at adulthood (Fig. 1b[Fig f1]c). Collectively, the brain transcriptomes show dynamic changes during development and sexual maturation, which are in general correlated with the known changes in behavioral, emotional, hormonal and cognitive processes^18^,^19^.

**Figure 2:**
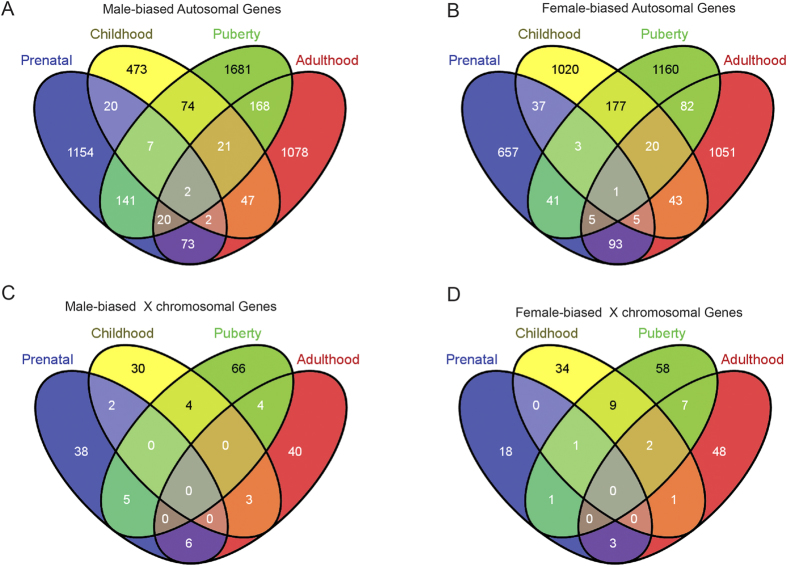
(**A–D**) Venn diagrams of sex biased genes at prenatal, early childhood, puberty and adulthood stages.

We compared the sex-biased genes in the brain among the four major developmental stages (Fig. 2). For genes located on autosomes, the male biased genes and the female biased genes often showed stage related changes, and only three genes remained the same pattern from prenatal time to adulthood (two for male biased genes, and one for female biased genes) (Fig. 2A,B). Interestingly, brains at puberty time show the largest number of sex biased genes, an indication of an important role of sex hormone during sexual maturation (Fig. 2A,B). For the X chromosomal genes, we observed a similar pattern and puberty time also show the largest number of sex biased genes (Fig. 2C,D). For the Y chromosome genes, we detected consistent expression of 12 genes in males at all four developmental stages (Supplementary Figure 1), including KDM5D, DDX3Y, ZFY, PCDH11Y, USP9Y, RPS4Y1, CYorf15B, TMSB4Y, NLGN4Y, UTY, EIF1AY and GYG2P, and four of them (DDX3Y, RPS4Y1, UTY and ZFY) are overlapped with six previously reported male biased genes using microarray^13^. In addition, there are also Y chromosome genes with stage specific expression, *i.e.* TBL1Y and SRY in prenatal time, PSMA6P1 and VDAC1P6 in early childhood, RNA45S5, NAP1, L1P2, ZFY-AS1, CTBP2P1 and ATP5JP1 in puberty time, and HSFY3P in adulthood (Supplementary Figure 1).

**Figure 3:**
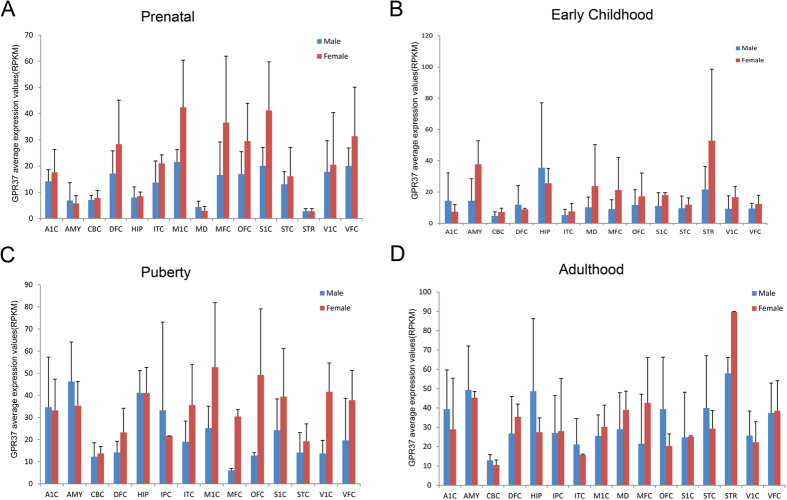
(**A–D**) GPR37 expression in female (red) and male (blue) brain regions at prenatal, early childhood, puberty and adulthood stages.

**Figure 4:**
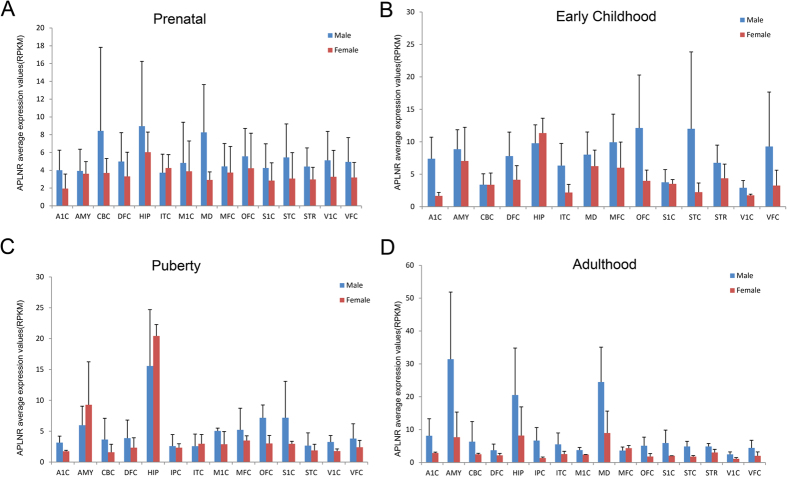
(**A–D**) APLNR expression in female (red) and male (blue) brain regions at prenatal, early childhood, puberty and adulthood stages.

There are two male biased genes and one female biased gene showing consistent pattern from prenatal stage to adulthood (Fig. 2A,B), suggesting that these genes may have essential function related to human male and female brain characteristics. Interestingly, two of them are G protein coupled receptors, including the female biased gene GPR37 (located on Chr 7) (Fig. 3), a key gene responsible for juvenile Parkinson’s disease^20^. Consistently, GPR37 was also a sex biased gene in mouse^21^, and the GPR37 knockout mice exhibited a sex biased phenotype that the aged GPR37−/− female mice showed increased anxiety and depression-like behaviors^22^. The other one is the male biased gene APLNR (on autosomal 11) (Fig. 4) that exhibited functional roles in the cardiovascular and central nervous systems^23^, as well as in glucose metabolism, embryonic and tumor angiogenesis and as a human immunodeficiency virus (HIV-1) co-receptor^24^,^25^. This gene also showed male specific down-regulation in the mouse brain when exposed to sub-chronic variable stress^26^. Another male biased gene is IL33 (located on Chr 9) (Supplementary Figure 2), acting as a chromatin-associated nuclear factor with transcriptional repressor properties, and also a reported candidate for Alzheimer’s disease^27^.

### Functional annotation of the sex biased genes

**Figure 5:**
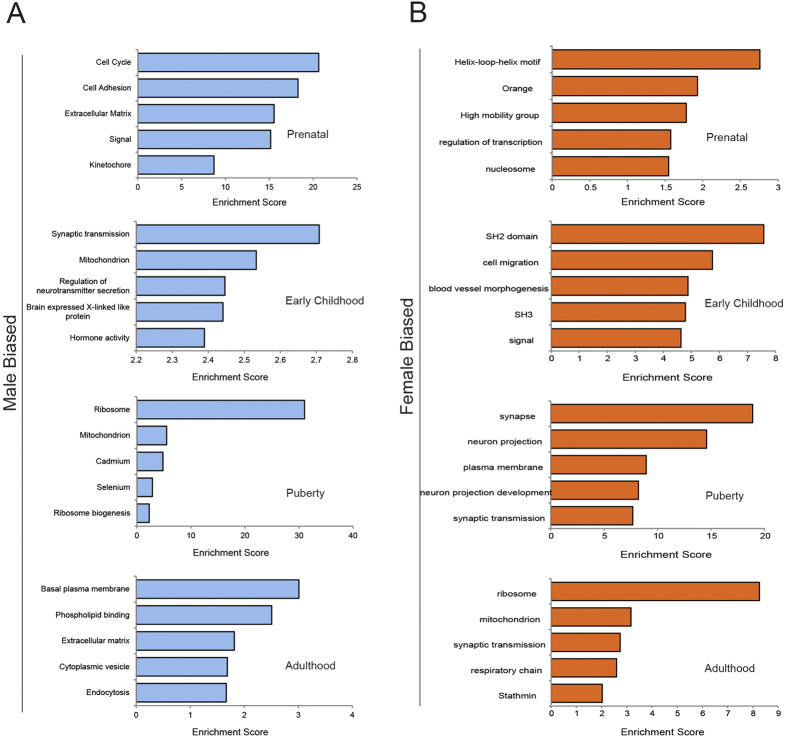
Functional annotation of the sex biased genes. (**A**) Function annotation of the male biased genes at prenatal, early childhood, puberty and adulthood stages. (**B**) Function annotation of the female biased genes at prenatal, early childhood, puberty and adulthood stages.

To explore the biological significance of the sex biased genes, we used the DAVID system to perform cluster functional annotation. For the enriched functional categories of the sex-biased genes, we observed a stage-specific pattern (Fig. 5 and Supplementary Table 9–16). At prenatal time, the highest enrichment score of the male biased functional category is cell cycle while the category for the female biased genes is transcription factor activities (Fig. 5A). As cell cycle genes are important for neurogenesis, this observation is consistent with the aforementioned speculation that the between-sex divergence of the brain starts as early as prenatal time^28^,^29^. At early childhood, the category of synapses is over-represented for the male biased genes (Fig. 5A and Supplementary Table 10). Synapses are also over-represented for the female biased genes, but at puberty time (Fig. 5B and Supplementary Table 15). Since synapse function is important for brain development and maturation, and many genes involved in synapse function are also risk genes for neurodevelopmental disorders like schizophrenia and autism^30^,^31^, the shifted stages of synapse function seems to be correlated with behavioral and emotional differences between males and females during brain development and sexual maturation^18^,^32^, although the observed pattern seem to contradict the traditional view that girls sexually mature 1–2 years earlier than boys. In contrast, we did not observe any functional enrichment at adulthood (Fig. 5).

### Enrichment of the sex biased genes involved in brain diseases

**Figure 6:**
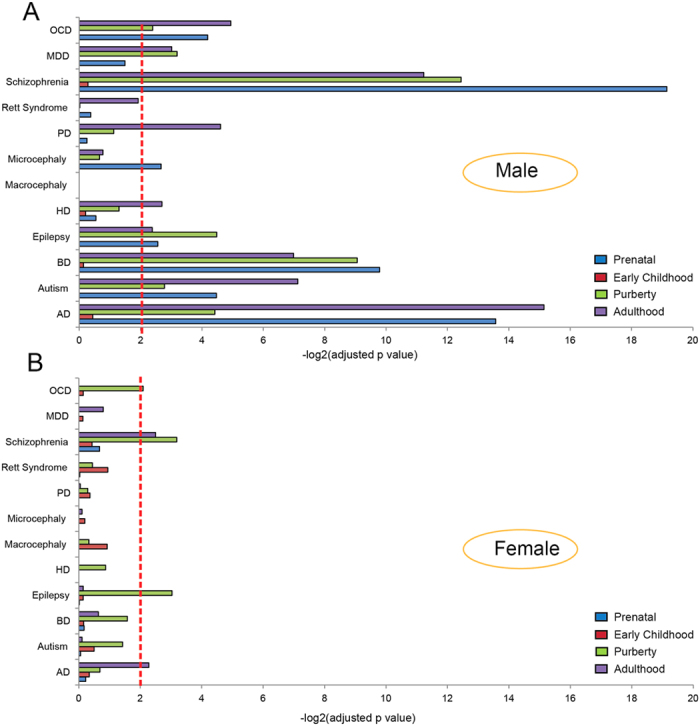
Enrichment scores of the sex biased genes for brain diseases. Dashed line indicates significance (corrected *p*-value < 0.01). (**A**) Enrichment of the male biased genes. (**B**) Enrichment of female biased genes. These brain diseases include OCD (obsessive compulsive disorder), MDD (major depressive disorder), PD (Parkinson’s disease), HD (Huntington’s disease), BD (bipolar disorder) and AD (Alzheimer’s disease).

We next assessed the enrichment of the sex biased genes previously implicated in various neurological and psychiatric disorders. We observed a sharp contrast between the male biased genes and the female biased genes. For the male biased genes, they are highly enriched for neurological and psychiatric disorders (autism, bipolar disorder, schizophrenia, Alzheimer’s disease and Parkinson’s disease) (Fig. 6A). A list of these genes is shown in supplementary Table 17. For example, during prenatal time, the significantly enriched brain diseases include OCD (obsessive compulsive disorder), schizophrenia, microcephaly, epilepsy, bipolar disorder, autism and AD (Fig. 6A). But for the female biased genes, only a few diseases were barely significantly enriched (OCD, schizophrenia, epilepsy and AD) (Fig. 6B). Interestingly, microcephaly, a neurodevelopmental disorder is only enriched for the male-biased genes during prenatal time which coincides the observed functional enrichment for cell cycle genes (Fig. 5A)^28^. Notably, no significant enrichment for any disorders was detected for early childhood when sex hormones are relatively inactive (Fig. 1c). Collectively, these findings suggest that sex differences of gene expression in the human brain are congruent with the well recognized differences in disease incidence between males and females^33^.

### Evolutionary divergence of the sex biased genes

**Figure 7:**
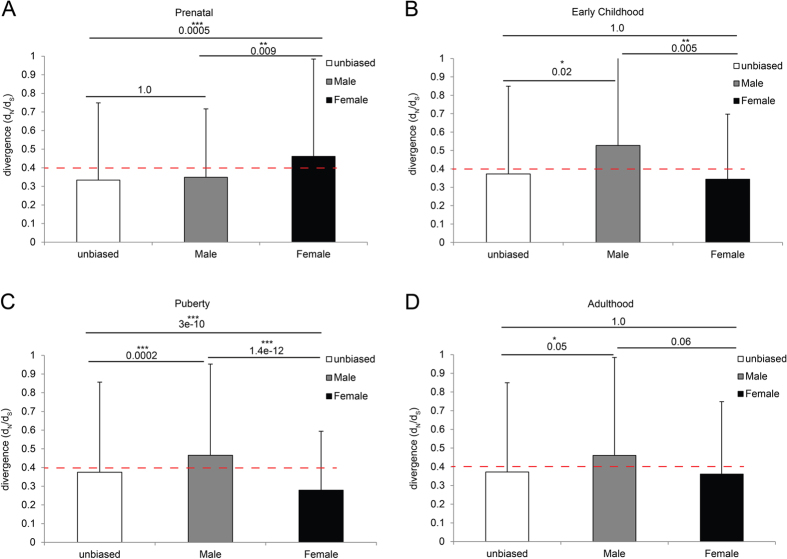
(**A–D**) Sequence divergence estimates of the male biased and female biased genes at prenatal, early childhood, puberty and adulthood stages.

Finally, to test whether natural selection would act differently on the male biased genes and the female biased genes, we examined sequence divergence data of human vs. chimpanzee orthologous in order to identify how sex specific selection shifts over the course of the life cycle. We estimated rates of sequence changes (non-synonymous substitution vs. synonymous substitution, dn/ds) for sex biased categories at the four developmental stages of the life cycle. Among the four developmental stages, three stages consistently showed larger dn/ds values for the male biased genes as compared with the female biased genes, suggesting that the male biased genes are relatively less constrained (negative selection) compared with the female biased genes (Fig. 7). Prenatal time showed an opposite pattern where the female biased genes have relatively larger dn/ds values (Fig. 7A), suggesting that sex specific selection pressures shift over the life cycle.

## Discussion

The transcriptome time series analyses presented here were built on the released human brain time series data from prenatal to postnatal stages (http://www.brain-map.org/), which suggests a complex picture of developmental stage dependent changes and shifted evolutionary pressures shaping sexual dimorphism of the human brain. Although sex dimorphic gene expression is common in the brain^3^, it is not uniform, and usually show brain region specific sex biased expression. In addition, some brain regions may have a higher burden of sex biased expression compared to the others. Notably, we demonstrate that the male biased gene expression likely have functional consequences relevant to human brain diseases, reflected by the enrichment of disease related genes (Fig. 6A), while no such pattern was observed for the female biased genes (Fig. 6B). This result is consistent with the “female protective model” in neurodevelopmental disorders^34^.

Our results indicate that the gene expression divergence underlying brain differences between males and females begins rather early in brain development, which was also observed in the animal studies (*e.g.* chicken and zebra fish)^11^. In general, the degree of sex bias increases over time after birth because the reproductively matured individuals require the largest contingent of sex biased genes. However, we should notice that prenatal time has more sex biased genes compared with early child stage, especially for the male biased genes because sex determination is accomplished during prenatal time. Puberty time has the largest number of sex biased genes for both male and female biased genes. Puberty time also plays an important role in postnatal brain development during sexual maturation^35^,^36^. It was reported that the mean volume of medial temporal lobe increases in adolescent boys but decreases in adolescent girls with the progression of puberty^32^.

Sex specific gene sequence divergence estimates can be used as a proxy for the sex specific evolutionary pressures acting on genes underlying dimorphic traits. Our analysis indicates that strong selection pressure exerted on female brain at puberty time. It is during this critical period that selection on female reproduction is expected to be intense. Clearly, sex biased gene expression patterns throughout the life cycle are complex, with distinct suites of genes controlling sexual differentiation in embryonic stages and sexual dimorphisms at adult stages. Similarly, the sex specific selection pressure also change its intensity from one sex to the other over the life cycle.

We also compared the list of sex biased genes in this study with a published list of 1,108 genes (adult brain) based on microarray data^14^, and only 150 genes were overlapped between them (Supplementary Figure 3), likely due to the differences in technical platforms, brain developmental stages and sample size. It should be noted that the sample size of this study is limited owing to the lack of large repositories of human post mortem brain tissues, which may potentially limit the statistical power. Therefore, it will be necessary to collect more brain samples at different developmental stages especially prenatal time in order to obtain a complete picture of brain sex dimorphism not only at gene expression level, but also at epigenetic regulations, *e.g.* DNA methylation and histone modifications^14^,^37^,^38^,^39^.

## Methods

### Data acquisition

All brain RNAseq data was downloaded from BRAIN SPAN (atlas of the developing human brain) (www.brainspan.org) covering the developing stages ranging from 8 post-conceptional weeks (pcw) to over 40 years of age and we divide the periods into 4 major developmental stages including prenatal(8–cw–24pcw), early childhood(4mos–4yrs), puberty(8yrs–19yrs) and adulthood stages(21yrs–40yrs).

### Sex biased gene expression analysis during human brain development

The RPKM (reads per kilo base per million) values were used as measurements for gene expression quantification, which allow accurate comparison between males and females. Differentially expressed genes were identified based on integer count data using DESeq R package^40^ which determines differential expression by modeling count data using a negative binomial distribution. First, size factors are calculated that take into account the total number of reads in different samples. Second, a dispersion parameter is determined for each gene which accounts for biological variation between samples. Finally, a negative binomial distribution is used to fit the counts for each gene. The p-value is calculated based on the fold changes. The *p* < 0.05 was consider significant.

### Gene ontology (GO) enrichment analysis

For the list of differentially mRNA genes, we tested weather each had enriched GO terms in biological process and molecular functions using the DAVID system^41^,^42^. Only those functional annotation terms associated with the various sets of differentially expressed genes were clustered that were significantly enriched (*p* value is smaller than 0.05) compared with the function annotation terms associated with the total population of genes expressed in at least one of the male or female samples.

### Disease enrichment analysis

To test whether the sex biased genes are enriched for brain diseases, we assessed their enrichment scores. Disease gene sets were download from the Genotator database^43^. Enrichment was tested using the hypergeometirc probability distribution functions in Excel. The population universe was set to 22,327. *P* values were corrected by applying the Bonferroni method using the p adjust package. Enrichment was only considered significant if the Bonferroni corrected *p* value was smaller than 0.01.

### Analysis of rates of molecular evolution for brain sex biased genes

Rates of human brain sex biased genes were estimated as the pairwise dn/ds between human and chimpanzee orthologous genes. The sequence data was from the Ensemble database (Ensembl genes 78, GRCh38). Pairwise *t* test was used to evaluate differences of dn/ds values among different gene sets or between the male biased genes and the female biased genes. *P* values were corrected by applying the Bonferroni method using the p adjust R package. Enrichment was only considered significant if the Bonferroni corrected *p* value was smaller than 0.05 Table 2.

## Additional Information

**How to cite this article**: Shi, L. *et al.* Sex Biased Gene Expression Profiling of Human Brains at Major Developmental Stages. *Sci. Rep.* **6**, 21181; doi: 10.1038/srep21181 (2016).

## Supplementary Material

Supplementary Information

Supplementary Dataset 1

Supplementary Dataset 2

Supplementary Dataset 3

Supplementary Dataset 4

Supplementary Dataset 5

Supplementary Dataset 6

Supplementary Dataset 7

Supplementary Dataset 8

Supplementary Dataset 9

Supplementary Dataset 10

Supplementary Dataset 11

## Figures and Tables

**Table 1:** Information of human brain samples used in this study.

Developmental stage	Age range	Male samples	Female samples
Prenatal	8pcw–24 pcw	4–9	4–8
Early Child	4mos–4yrs	3–6	2–3
Puberty	8yrs–19yrs	2–4	2–3
Adult	21yrs–40yrs	2–3	2–3

^*^pcw (post conception weeks), mos (month), yrs (years). Detailed sample information can be obtained from BRAIN SPAN (atlas of the developing human brain) (www.brainspan.org).

**Table 2:** Differentially expressed genes between males and females during human brain development.

Developmental stage	Male biased genes	Female biased genes	Total
Prenatal	1,573	1,003	2,576
Early Child	761	1,554	2,315
Puberty	2,408	1,756	4,164
Adult	1,785	1,444	3,229
