# Supplementary material for: Sex Biased Gene Expression Profiling of Human Brains at Major Developmental Stages
Source: Sci Rep. 2016 Feb 16;6:21181. doi: 10.1038/srep21181 (PMC4754746; doi:10.1038/srep21181)
Supplement: Supplementary Information [file srep21181-s1.pdf]

Supplementary Information

Sex Biased Gene Expression Profiling of Human Brains at Major Developmental Stages

Lei Shi[1,2,3,4,§], Zhe Zhang[1,2,3,§] and Bing Su[1,2,3*]

[1]Faculty of Life Science and Technology, Kunming University of Science and Technology, Kunming 650500, China;

[2] State Key Laboratory of Genetic Resources and Evolution, Kunming Institute of Zoology,

Chinese Academy of Sciences, Kunming 650223, China;

[3] Yunnan Key Laboratory of Primate Biomedical Research, Kunming 650000, China.

[4] The Molecular & Behavioral Neuroscience Institute (MBNI), University of Michigan, 109 Zina

Pitcher Place, Ann Arbor, MI 48109-2200, USA

Figure S1. Venn diagrams of sex biased genes on Y chromosome at prenatal, early childhood, puberty and adulthood stages.

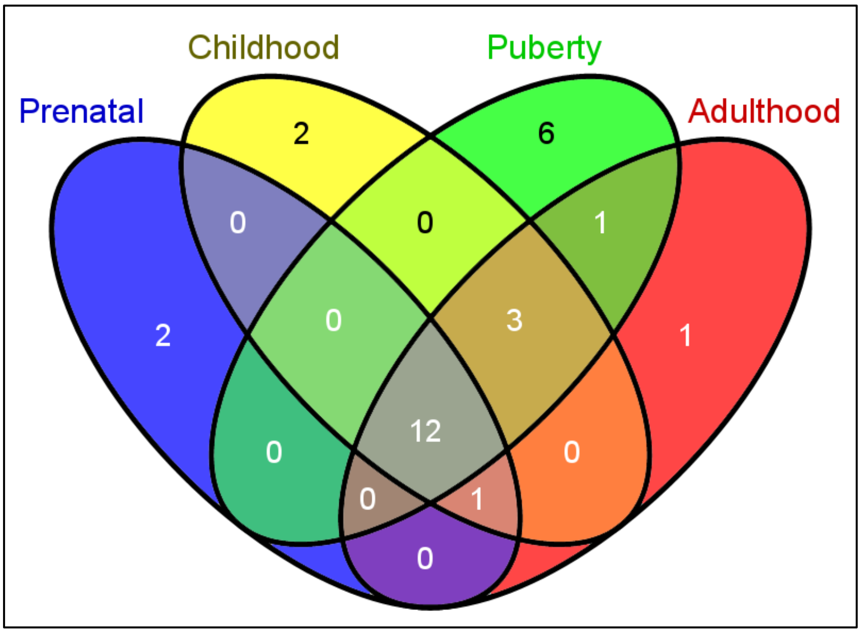

Figure S2. Il33 expression in females (red) and males (blue) in the human brain regions at prenatal, early childhood, puberty and adulthood stages.

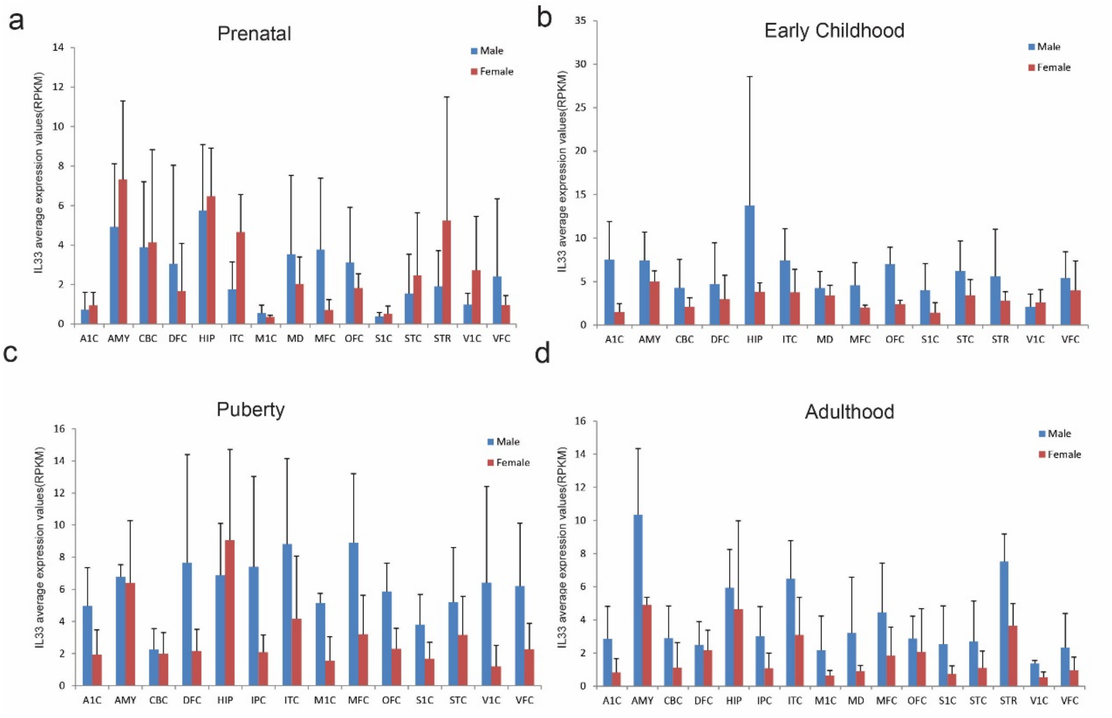

Figure S3. Venn diagram of sex biased genes based on comparison between the gene list of this study (yellow pie) and the published dataset-HMG[1] (blue pie).

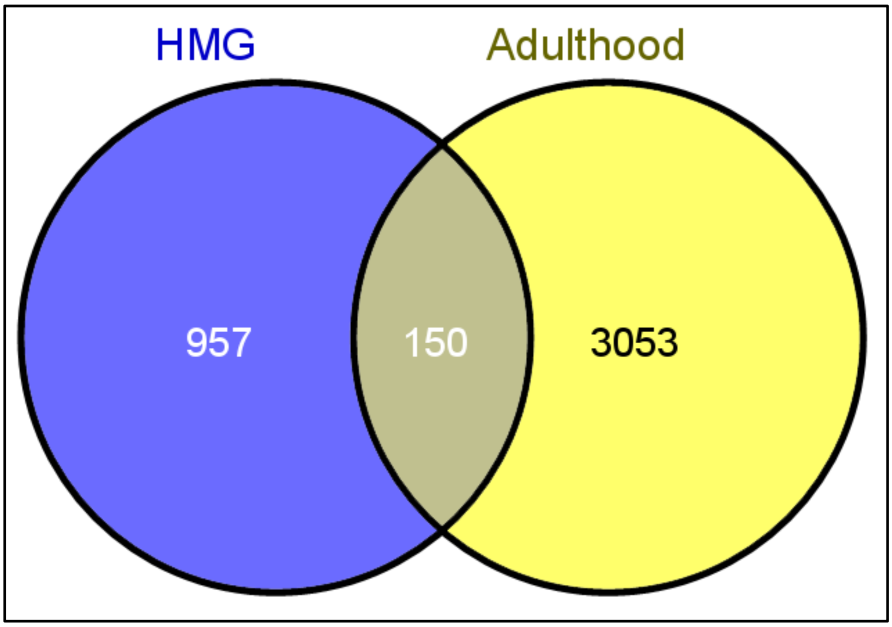

References

1. Xu, H. *et al.* Sex-biased methylome and transcriptome in human prefrontal cortex. *Hum Mol Genet* **23**, 1260-70 (2014).
